# What safety can we talk about when all we see is difficulty? The impact of geographic and cultural determinants of unintentional injury in rural and mountainous Mugu, Nepal

**DOI:** 10.3389/fpubh.2025.1599047

**Published:** 2025-06-12

**Authors:** Katherine Weatherburn, Divya Parmar, Puspa Raj Pant, Monila Parajuli, Jamie Murdoch

**Affiliations:** ^1^Department of Population Health Sciences, King's College London, London, United Kingdom; ^2^Centre for Public Health and Wellbeing (CPHWB), University of the West of England, Bristol, United Kingdom; ^3^Independent Researcher, Kathmandu, Nepal

**Keywords:** primary caregivers, determinants, gender, perceptions, physical environment, rural, Nepal, unintentional injury

## Abstract

**Introduction:**

Unintentional injuries are a significant health concern in rural Nepal, where geographic isolation and limited healthcare infrastructure exacerbate their impact. In the remote district of Mugu, primary caregivers of children navigate complex physical and social landscapes daily. This study explores how primary caregivers perceive the determinants of injury. By examining the interplay of environmental, social, and infrastructural determinants shaping injury risks, the findings provide insights into the unique challenges of injury in rural settings.

**Methods:**

In 2017, seven focus group discussions were conducted with 56 participants (95% female) in Mugu, Nepal. Using Braun and Clarke's thematic analysis framework, transcripts were analysed inductively, using NVivo 14 to assist with coding and identification of themes.

**Results:**

Five themes were identified; “Precarious environment,” “Limited choices due to life precarity,” “Gendered labour roles,” “Perceived control over injuries,” and “Healthcare access and quality,” with environment and gender underpinning all themes. Unintentional injuries were normalised, and outdoor environmental risks perceived as beyond primary caregivers' control. Conversely, some agency was expressed in home safety practices. Fatalistic beliefs served as coping mechanisms. Barriers to healthcare access led to reliance upon traditional remedies, adversely affecting injury outcomes.

**Discussions:**

Environmental, gender and socio-cultural factors shape injury patterns and prevention opportunities in Mugu. Primary caregivers face constrained options for prevention. However, local, municipality level policies which are socio-culturally relevant and tailored to meet caregiver needs, alongside infrastructure improvements, provide an opportunity to mitigate injury risks and also reduce associated socioeconomic impacts.

## 1 Introduction

The percentage of Nepal's rural population is considered to be the highest in South Asia (1), with 60% of its local administrative areas designated as Rural Municipalities (2). In 2018 it was estimated that more than three-quarters of the population (78%) lived in rural locations (3). Although the recent Census (4) recorded the urban population as 66%, it is considered an exaggerated estimate (2). Rural populations are generally considered to be of lower socioeconomic status and often perceive and respond to risks differently than those from higher socioeconomic backgrounds (5). Research has identified that social factors like income and education influence risk perception, with lower socioeconomic groups often underestimating certain risks due to lack of education and awareness (6).

People in lower socioeconomic groups are often more exposed to physical and environmental hazards of injuries (7). Injury is defined as “unintentional or intentional damage to the body resulting from acute exposure to thermal, mechanical, electrical, or chemical energy or from the absence of such essentials as heat or oxygen” (8). Unintentional injuries, which is the focus of this study, are a significant global public health concern and a leading cause of morbidity and mortality in high income countries (9), as well as in in low-and middle-income countries like Nepal (10, 11). The WHO STEPS survey found that people living in rural municipalities had more injuries (11.74%) compared to people living in big cities (9.67%), and 10.89% living in peri-urban towns (12). According to the Ministry of Health and Population (MoHP) 2024 Report (2079 BS)^1^, there were over 109,000 cases of injuries in health facilities in the Karnali province, a mountainous region of Nepal where Mugu district is located. The majority of these injuries (80%) were identified as “fall, fractures and injuries” (13). A hospital-based study conducted in Karnali Academy of Health Sciences recorded 460 injured patients, 42.2% of which injuries were caused by falls (14). In addition, the Karnali province had the second highest percentage of burn injury patients (8%) in Nepal (13).

Social determinants, including employment and living conditions contribute to higher exposure to occupational and environmental risks in low-income communities (15). In addition to exposure to injury, this population may have less access to reliable health information, leading to a reduced awareness of potential risks (16). This can limit their ability to make informed decisions about prevention of potential injuries to children (17), and also seeking medical attention post-injury (18, 19). Facing barriers to mitigating risks, such as limited access to health services, social support, and financial resources are limitations which affect their ability to recover from risks or injuries (20, 21). Trust in healthcare systems and services is often lower among individuals from disadvantaged backgrounds (22), and living in areas with limited healthcare access can lead to avoidance of medical treatment, particularly in underserved populations in Nepal (23).

However, the specific social determinants underlying the patterns of unintentional injury in remote and rural Nepal remain sparsely researched. This study therefore critically analyses primary caregivers (PCGs) perceptions of unintentional injury in Mugu. Analyzing discussions of participants offers a unique opportunity to gain insight into social determinants that contribute to injury patterns (24). Understanding participants' perceptions provide valuable insight into these social determinants of injury (19), and potential entry points for targeted interventions for the prevention of subsequent injuries can be identified (25).

## 2 Methods

### 2.1 Study setting and design

This study took place in Mugu, a remote mountainous district in Mid-Western Nepal, which according to the Department of Roads, has only 27 km of earthen-surfaced roads, connecting Bulbule and Gamgadhi. In the Nepal Census 2021, Mugu had a population of 64,550 (86% below the age of 50 years, including 24% below 10 years) with a population density of just eighteen, compared to 198 per square km nationally. 61% of households have five or more members, and one quarter of the population over 10 years is married before the age of 18 years. 31.3% of the population age five or more is not able to read or write compared to 23.3% nationally (4). Mugu has a poor health service delivery record (26), and 2.73% of Mugu's population are identified with a disability compared to 2.25% nationally.

The mountainous terrain significantly influences home architecture, with many houses being multi-level and built on slopes due to the resulting prohibitive cost of building on flatter land. Over 60% of houses are made up of thatch, timber, mud or slate, and ~40% of households do not have access to any type of piped water. Over 90% of households use firewood or kerosene for cooking energy. Subsistence farming is the primary livelihood, however, the rugged landscape means only 5.2% of land is cultivable (27). This geographical feature and extreme drought conditions directly contribute to food insecurity, reflected in the majority of households in Mugu facing acute food shortages for much of the year (28).

**Figure 1:**
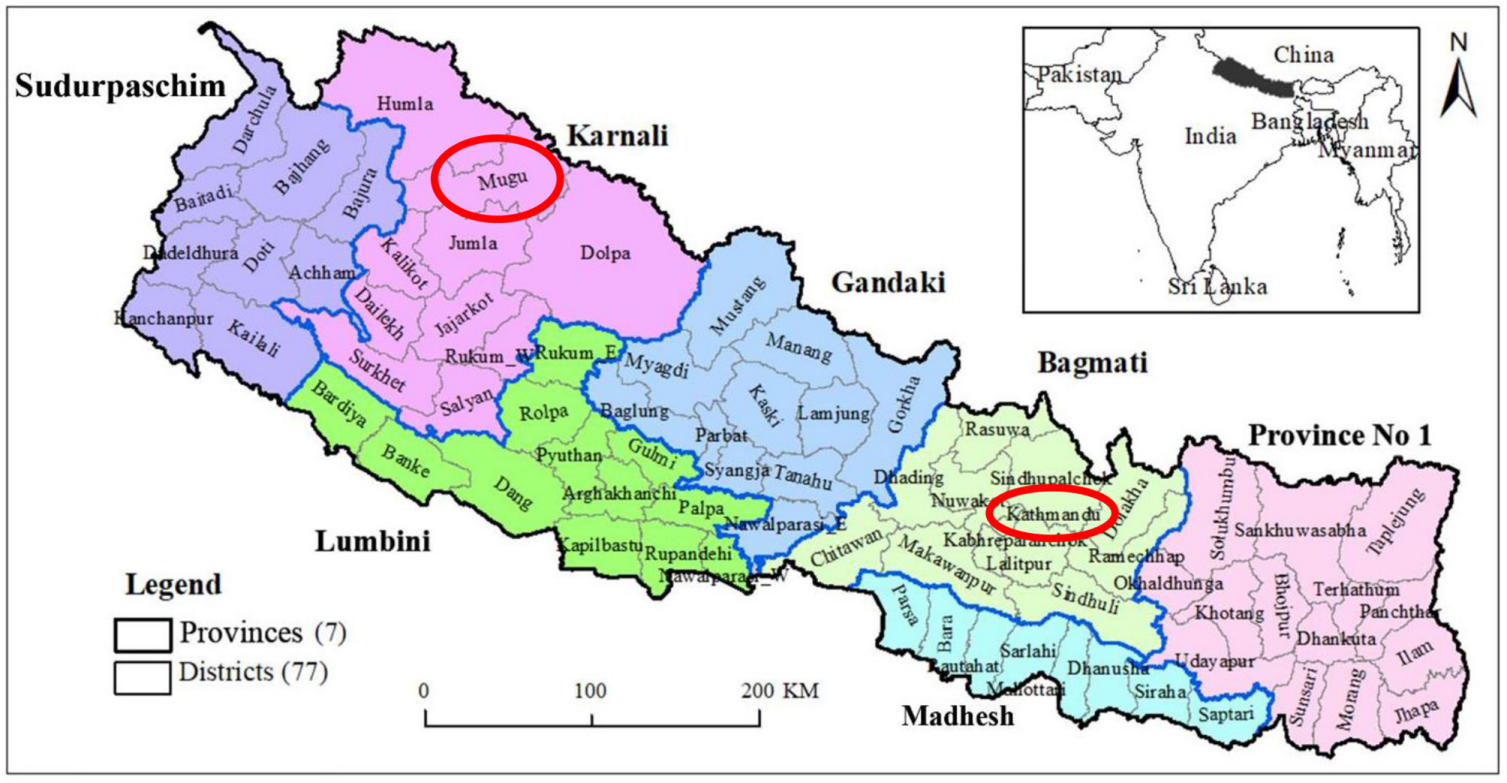
Map of Nepal showing provinces and districts, including Mugu and Kathmandu. Adapted with permission from “Location map of Nepal showing the seven provinces and 77 districts” by Bipin Kumar Acharya Samir Kumar Adhikari, Shreejana Pandit, Basanta Kumar Neupane, Binod Kumar Paudel and Laxman Khanal, licensed under CC BY-SA 4.0.

Map of Nepal showing provinces and districts, including Mugu and Kathmandu. Adapted from Acharya et al. (49) under the Creative Commons Attribution-ShareAlike 4.0 International License (Figure 1).

This study was part of a larger project about safety and injury which the first author led as an external researcher for a UK Aid funded road infrastructure project. This qualitative research study, using focus group discussions (FGDs) was guided by an interpretivist epistemology. This approach implicated assumptions that participant's sensemaking about injuries and their determinants were socially constructed through their interactions with each other and the world around them. This approach also recognizes that reality is experienced differently by each person, depending on their context, social position, and circumstances (29). Given this perspective, the use of FGDs was felt to be crucial to gain a deeper understanding of injury from the perspective of PCGs in Mugu. This method was deemed appropriate in this under-researched area, as FGDs provide an opportunity to develop an understanding about people's thoughts and beliefs and why they think the way they do (30). To facilitate this, a semi structured framework and FGD topic guide about safety and injury was developed by the first author, in conjunction with the research assistant and co-author. This was piloted by MP with PCGs in Mugu prior to the finalization of the guide. Topics included the contextual and environmental determinants of injury, injury type and prevalence, factors which increased risk of injury, including place and context of occurrence. Topics around safety and injury prevention were discussed in terms of beliefs about injuries, whether they were thought to be preventable, and beliefs about treatment for injuries.

To be included PCGs needed to be residents of Mugu and were the primary caregiver of at least one child aged 10 years or below. Fulfilling the inclusion criteria of a PCG, participants were selected through non-randomized convenience sampling strategy. Participants were identified from nineteen villages which were within a 2 h walking distance of the central settlement of Gamgadhi in Mugu, which was based upon a mapping exercise conducted by Mugu based research assistants. As advised and facilitated by Mugu based research assistants; to promote group dynamics and increase participant comfort, participants were grouped according to geographic proximity as well as similar socioeconomic and cultural backgrounds. FGDs were held in enclosed private spaces such as community centers.

The first author was the primary facilitator for the FGDs was supported by MP who also acted as interpreter and note taker. MP, a native Nepali speaker, translated verbatim from English to Nepali, and Nepali to English. Notes were taken in Nepali. Particular attention was given to the phrasing of questions, favoring the use of open-ended questions, using neutral statements, probing techniques, and repeating questions if needed. The first and four author gave adequate time for pauses in answers from participants, repeated or echoed responses to ensure correct understanding, and sought clarification when required. FGDs were conducted until the same perspectives were repeatedly expressed, which occurred after seven FGDs. With written informed consent, the FGDs were audio recorded to enable subsequent transcription and translation. Each FGD lasted between 1 and 2 h.

In total, 7 FGDs comprising groups of 7–11 participants (53 participants), aged 19 to 60 years, were conducted in June 2017. Participants reported having multiple roles including construction, business, and subsistence farming. All participants were Nepali and identified as being a PCG of a child or children aged 10 years and below. The majority of participants (95%) were female, which can be said to be reflective of the sex of PCGs in Mugu.

Ethical approval was obtained from King's College London, Ethics Reference Number LRS-16/17-4111. All audio recordings and transcripts were password protected. Data were only available to the primary researchers involved in this research study. Any identifiable information was removed during transcription to maintain participant confidentiality.

### 2.2 Analysis

**Figure 2:**
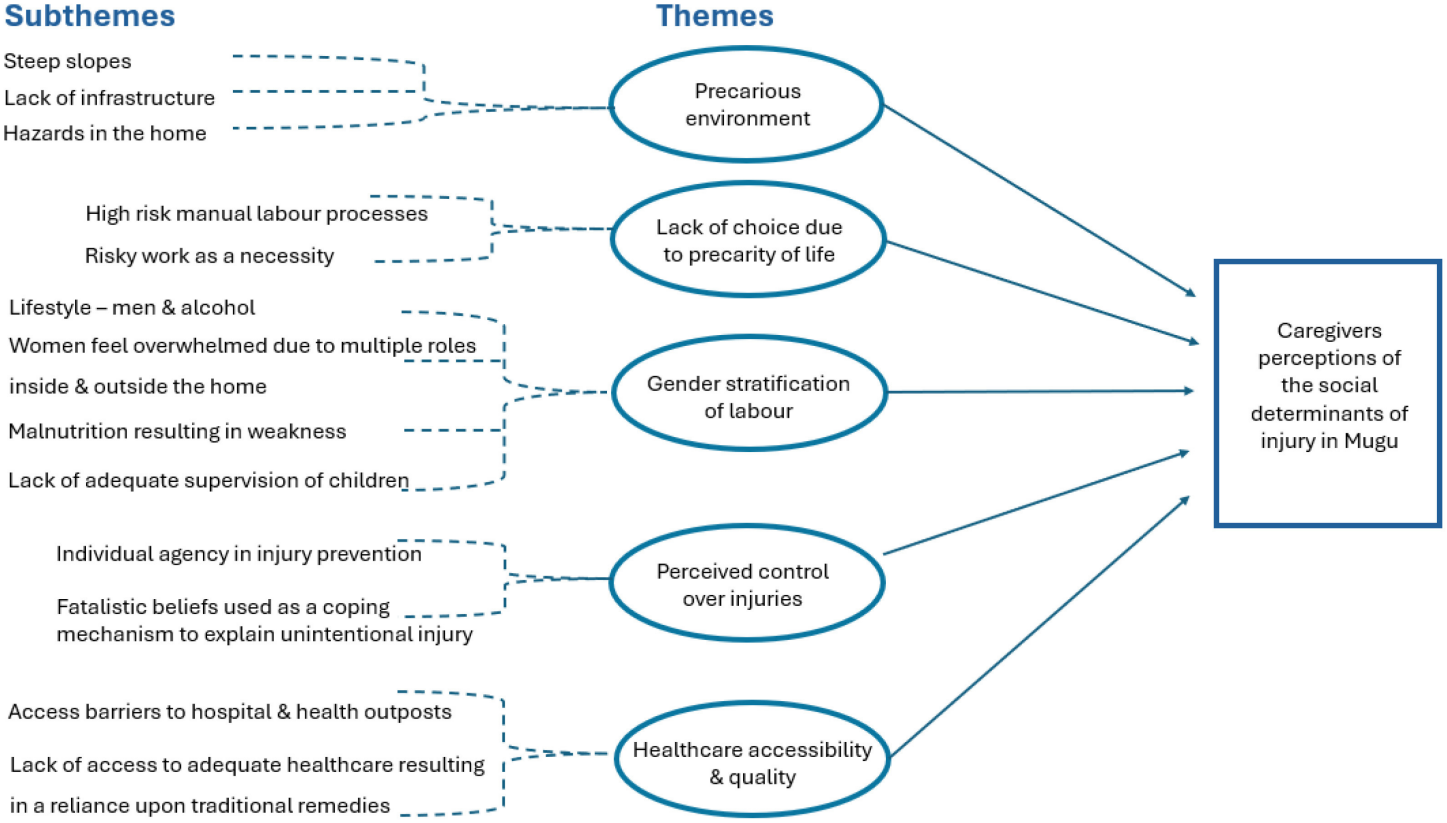
Thematic map.

Analysis of FGDs was a multi-step process incorporating strategies for reflecting on and triangulating different perspectives shared by FGD participants and interpretations within the research team. Firstly, immediately following each FGD, the first and fourth author, and research assistants present, shared their perspectives of the main issues discussed in the FGD. These debrief sessions focused on identifying major themes, notable ideas, and any points requiring clarification or further exploration. This enabled real time comparison and refinement of findings developed by the research team. This collaborative step promoted a shared understanding and interpretation, important given the need for translation during the FGDs, but also for giving a voice to local researchers in how to interpret the FGD data. Secondly, practical and logistical aspects of the FGD, such as ways to improve facilitation, were also discussed to enhance the quality of subsequent FGDs. In addition to the research team's reflections, Mugu based research assistants gathered informal feedback from some participants regarding their experiences of the FGDs, gaining insight and suggestions for improvement for subsequent FGDs. Thirdly, FGDs were then transcribed and translated into English and imported into QSR NVivo 2.0 software. Back translation and spot checks were conducted to promote data integrity. Fourthly, In 2024, coding based upon Braun and Clarke's (50) inductive thematic analytical approach was conducted by the first author, with the aim to reach theoretical saturation of themes. This required KW to acknowledge the time elapsed since original data collection, and how different determinants may or may not have endured and continue to shape perceptions of injury in rural Nepal today. Initial codes were then discussed with co-authors JM and DP, enabling the identification of subthemes, which were grouped together to create higher order themes (Figure 2). These themes were discussed with co-authors MP and PP who were able to speak directly to how these findings relate to injury prevention in Nepal.

### 2.3 Reflexivity

Reflexivity was a key consideration in this research, allowing for thoughtful examination of how the first author, a White British Female's positionality influenced and shaped the study. The first author's background as a female researcher from the UK, with previous experience in injury prevention and international development, undoubtably shaped both the design and implementation of this study. Her outsider status in the rural Nepali context brought with it certain assumptions, which she actively sought to interrogate throughout the research process. These assumptions were that PCGs may not be acutely aware of some of the injury risks involved in their daily tasks and activities, or that leaving children without adequate adult supervision could increase their risk of injury. Her position as an external researcher and collaborator within a UK Aid funded infrastructure project also presented inherent power dynamics which could influence power dynamics and participant responses during FGDs. Therefore, the involvement of research assistants, including those from Mugu, was integral to grounding the research in local cultural and social realities. Their local knowledge and understanding were crucial in identifying cultural nuances and potential biases, ensuring that the research approach was culturally sensitive and reflective of participants' lived experiences. These collaborations were essential for designing a research approach that respected participants' priorities and rhythms of daily lives, including aligning FGDs with farming schedules and meal times. By incorporating local knowledge and perspectives, the study sought to move beyond external assumptions of the first author, and reflect the lived experiences of participants.

When conducting this study, the first author had 8 years' experience in the international development sector, with a background that included work on injury prevention in Nepal. She brought a depth of understanding of the complexities of cross-cultural research. This prior knowledge of Nepal's socio-cultural and geographical context contributed to her understanding of the challenges faced by caregivers in Mugu, but also required her to actively and continually question her own assumptions. She brought a depth of understanding of the complexities of cross-cultural research, but remained aware that her perspective could never fully represent the views and experiences of the participants. She engaged in reflexive practices, including active listening and fostering respectful dialogue to create an environment where participants felt comfortable sharing their views. This reflexive approach aimed to balance the research process as much as possible and prioritize the authentic representation of participant voices within the study's findings, ensuring they were not overshadowed by the researchers' own agendas.

## 3 Results

As shown in Figure 2, 13 subthemes were identified during analysis, which were consolidated into five higher order themes, reflecting PCGs perceptions of the social determinants of unintentional injury in Mugu.

### 3.1 Precarious environment

The theme of “Precarious environment” highlights the remote and mountainous landscape of Mugu, where the challenging terrain serves as a fixed physical backdrop and omnipresent determinant of injury. This precarious landscape underpins and intersects with all other identified themes, shaping injury hazards faced by people on a daily basis.

#### 3.1.1 Steep slopes

Participants in all 7 FGDs discussed the precarious physical environment, the steepness of slopes as being hazardous and the cause of falls, particularly when working:

“*When we go to cut grass. When we go to graze cattle. The steep areas are always dangerous (due to very high altitude and narrow walking ways). Sometimes in these steep areas, we get dizzy (while walking) and fall.” (FGD 1)*

They also discussed the frequency and severity of associated falls, with some participants describing fatal outcomes:

“*We have to go to steep land so falls are common and people die (from injuries). Many people die from falls.” (FGD 3)*

Participants identified rain as a factor which increased the risk of falls from steep slopes:

“*The bad weather stones (rocks and debris) cause falls, heads get injured; landslide prone areas are also there. Some people trip on stones (rocks and debris) causing fall and get injured.”* (FGD 1)

However, although participants were able to identify inclement weather as a factor which further increased the risk of injury, they spoke about the unpredictable nature and lack of control they had over it:

“*We cannot control landslides, neither can we control floods. We are in so much risk.”* (FGD 5)

This highlights that participants are aware of the injury risks steep slopes present, and they are able to identify exacerbating factors such as inclement weather which can further increase risk of injuries from landslides and rockfalls. However, participants explained that it was necessary for them to interact with the precarious environment on a daily basis, and that they were helpless to prevent injury.

#### 3.1.2 Lack of infrastructure

Participants discussed their challenges navigating the rugged terrain. They identified a lack of good ‘flat' or ‘cemented' roads, paths and foot trails, which in conjunction with rain, was identified to be a cause of falls:

“*There are no cemented paths so we slip. Especially when it rains, the way gets muddy, (brings debris) and is (become) very slippery and we fall.”* (FGD 5)

Participants identified the mountainous terrain as a cause of falls:

“*It is not flat like the Terai so anyone can fall here.” (FGD 3)*

The terrain in conjunction with a lack of lighting was highlighted to be a cause of falls:

“*The paths are just not good here. In the Terai, there are lights on the way but here there are no lights on the foot trails so people fall.”* (FGD 5)

Participants compared Mugu to the Eastern flat plains of the Terai to emphasize their risk of injury, both in terms of landscape, and also better physical infrastructure in the Terai which could reduce the risk of injury. In addition, urban areas were also believed to reduce injury risk due to a reduction of manual processes:

“*Even doing a simple thing like cooking we get (burns and scald) injured. We won't get an injury if we stay still. Not like Kathmandu, here we have to grind, cut, and collect fodder so there is high chance of injury here.” (FGD 3)*

#### 3.1.3 Hazards in the home

Participants identified the home environment as being hazardous to themselves and the children in their care, with ladders and rooftops a source of injuries to children:

“*Children fall off the ladder as the ladder is steep, and narrow.”* (FGD 4)

The vernacular architecture which is typically flat rooftops without barriers, were identified by participants as hazardous to children and a known cause of falls:

“*My granddaughter fell from the rooftop and broke her hand.”* (FGD 2)

Participants said that “We” (females) are suspectable to burns while cooking, attributed to floor level cooking and the use of an open fire or rudimentary stove:

“*Our cooking methods are such that we get burnt a lot.”* (FGD 4)

### 3.2 Lack of choice due to precarity of life

Due to economic instability and need to survive, participants are forced to participate in high risk, labor intense manual processes which put them at high risk of injury, reflecting the poverty and resulting constraints they live in.

#### 3.2.1 High risk manual labour processes

Participants described the agricultural economy of Mugu, which included the need to interact with livestock. Due to laborious manual processes and rudimentary equipment used, this resulted in high risk of injury:

“*For our livelihoods we have to do work. One of my cousins went to feed the mule and the mule kicked him and he got injured. He was taken to hospital.”* (FGD 3)

Collecting fodder, or food for livestock using rudimentary hand tools, referred to by participants as ‘grass cutting', was commonplace. This practice predominately done by women using a sickle without protective gloves was said to cause cuts:

“*When cutting grass, our hands get cut due to sickle use.”* (FGD 3)

Sickles were also mentioned to cause cuts during food preparation, as was the traditional knife “Chulesi^2^”. Using other hand tools including a mortar and pestle were also said to result in injury:

“*While cooking (preparing food) you might get cut by a sickle. While making dal we need to mash dal so our fingers might get crushed. Chulesi and sickle might cut our fingers.”* (FGD 1)

Participants discussed that both the process of collecting firewood and chopping firewood could result in injuries:

“*When we go to collect firewood we get injured.”* (FGD 3)

“*We have to use an ax while chopping firewood and we get cuts. Cuts mainly on our hands, legs, face.”* (FGD 3)

#### 3.2.2 Risky work as a necessity

Participants emphasized their daily exposure to risk while performing domestic chores, and their lack of choice to avoid identified risks:

“*During working time we will get injured. We have to work in whatever condition it is.”* (FGD 2)

Although participants recognized the inherently hazardous nature of their work, they expressed that working in known hazardous conditions was essential for their survival:

“*Without working we don't get to eat.”* (FGD 6)

This was further highlighted when participants expressed feeling fear when carrying out necessary tasks:

“*Some people are scared of climbing trees^3^ and going to steep land. Some people climb trees even though they know it is dangerous, but they have to.” (FGD 1)*

Another participant highlighted their lack of choice but to participate in known high risk activities:

“*What safety can we talk about when all we see is difficulty. We need to walk long [distances] to collect water. Who is going to give us safety when we have to work all day?” (FGD 7)*

According to Census 2021, only 17% of households in Mugu have access to piped water, meaning 83% of households have to walk varying distances to collect water along perilous paths and foot trails.

### 3.3 Gender stratification of labour

Participants discussed societal dynamics, gender roles and lifestyle choices in relation to injury, highlighting how these factors intersect with daily tasks to influence injury experience.

#### 3.3.1 Lifestyle—men and alcohol

Participants identified alcohol use as a common cause of people falling from steep slopes:

“*Alcohol and the difficult terrain is the reason. Lots of injuries are due to alcohol consumption.”* (FGD 5)

They identified a combination of risk factors that could result in serious injury or death:

“*some people drink alcohol and fall and die.”* (FGD 3)

Participants discussed that it was predominately men who drank alcohol in Mugu:

“*Women might drink. But mainly it is men who drink and fall.”* (FGD 5)

#### 3.3.2 Women feel overwhelmed due to multiple roles inside and outside the home

Participants spoke of the numerous roles women have both inside and outside of the home, and identified that:

“*The overload of work is also the reason for injury.”* (FGD 4)

Women highlighted the risk of injury they encounter when collecting food for livestock, food preparation and cooking, water collection, construction work, and looking after children:

“*When going to the mill, plowing the field and cutting grass. When we go to get the grass we fall from the hill.”* (FGD 5)

They emphasized the burden of these multiple roles and responsibilities, resulting in stress and tension:

“*We women are weak and cannot think broadly as we have our children's tension, tension of household works. We worry if our child will get burnt, or use sharp objects, or if the cooker explodes.”* (FGD 4)

As a consequence, women described how they rushed different tasks and attributed this as a direct cause of injuries:

“*We rush to finish work so injury happens.”* (FGD 5)

“*Because we are always in rush. When we are cutting vegetables we get cuts. We get burnt on our hands and legs.”* (FGD 3)

#### 3.3.3 Malnutrition resulting in weakness

Some participants identified malnutrition during pregnancy as a factor that leads to weakness and feeling dizzy, contributing to injuries:

“*We are very poor. We didn't get enough care when we had to bear children and that's why our bodies are weak now and we feel dizzy.”* (FGD 5)

They also stated that having multiple children and insufficient nutrition led to weakness and injury:

“*Weakness also causes injury. We have many children and we don't get to eat enough or often enough. That's why our body is weak while working, so we get dizzy, fall and get injured.”* (FGD 4)

Participants expressed their concern of falling while carrying children, and the risk not only for themselves but also their children:

“*we have to carry small children on our backs. If we fall then the small children might also fall with us. (FGD 4)*

#### 3.3.4 Lack of adequate supervision of children

Participants discussed that they leave children in the care of their older siblings, an arrangement for child supervision they identified as being inadequate and resulting in injury:

“*We leave our older children to look after the younger one. So there is a high chance of the younger child falling off the roof.”*(FGD 4)

Participants also reported that children fell onto the floor level stove during lapses in supervision, resulting in burns:

“*Once I was making roti, when my baby fell on the hot stove.”* (FGD 4)

Although participants identified that inadequate supervision of children could result in child injury, participants discussed the quandary they faced with regards to supervision of children, and their need to leave children unsupervised when they go out to work. They also discussed that

“*If we had good jobs and didn't need to go to the field or to raise cows then we could take our children to school ourselves and then they would have no injury. We have to work because we don't get enough to eat without working. Therefore we have to leave our children all alone at home.” (FGD 4)*

### 3.4 Perceived control over injuries

Participants shared varying perspectives on their ability to prevent injuries. Some participants felt that they had individual control, whilst others argued that, due to external factors, injuries were inevitable.

#### 3.4.1 Individual agency in injury prevention

Some participants expressed that they had an ability to prevent injury, in the form of prioritizing caution and avoiding particular outdoor environments which they identified as risky:

“*Yes injuries can be prevented. We need to be careful. We should not walk in those areas where trees might fall or where stones might fall. We need to be careful.”* (FGD 2)

However, when being in such contexts was deemed unavoidable, participants said that they should prioritize safer behaviors over their own comfort in an attempt to reduce risk of injury:

“*Our backs hurt when we walk for too long, but we also can't sit in some places because there is a chance of being hit by a falling trees (rocks or wild animals).”* (FGD 6)

As discussed, environment was emphasized to impact an individual's agency to control or prevent injuries. However, in contrast to the outside environment, participants positioned the home as a place where they were able to exert a level of control and reduce the risk of injury:

“*At home we can somehow prevent injuries. I make sure that I keep sharp objects high up, I make sure kids don't touch them. When we are in and around our house we have control over things and we can take precautions, but when we go to jungle or walk on (foot) trails if anything happens we have no control.”* (FGD 4)

#### 3.4.2 Fatalistic beliefs used as a coping mechanism to explain unintentional injury

Some participants attributed injuries as being out of their control and occurring as a result of karma and fate:

“*If it is fate then whatever it is [that we are doing] we might get injured.”* (FGD 2)

Others discussed their injuries as being a form of karma or punishment from God:

“*Let's say if someone took my land forcefully then I go to God, the spirit comes to them to take revenge [in the form of injuries].”* (FGD 1)

Additionally, fatalistic beliefs were discussed in relation to wounds not healing which was viewed as a punishment by witches and spirits:

“*Yes, if you do something bad to others then the spirit comes to us and the wound doesn't heal.” (FGD 1)*

### 3.5 Healthcare accessibility and quality

Participants discussed barriers preventing them from accessing quality healthcare which included geographical isolation, financial constraints and lack of available treatment and medical expertise in local health facilities.

#### 3.5.1 Access barriers to hospital and health outposts

Participants identified distance and geographical factors as barriers to receiving hospital or health outpost treatment:

“*We have to take the injured person to the hospital in a vehicle or stretcher. When we get to the hospital there are not a lot of medicines there.”* (FGD 4)

In addition to a lack of medicine, a lack of medical expertise, particularly at nighttime was also discussed as a barrier to receiving treatment:

“*Recently my brother had been taken to hospital as he got kicked by mule. He was taken to hospital at night and then referred to the hospital in Nepalgunj.” (FGD 3)*

For this participant, the hospital advised them to travel to another district several days away to access appropriate healthcare. The associated costs of transportation, accommodation, and food for both the carer and patient, combined with the lengthy delay in receiving the correct treatment, significantly exacerbated the burden. Participants highlighted that these challenges created additional financial barriers to accessing quality medical care.

“*to treat those injuries we take other loans and the loans piles up.” (FGD 5)*

#### 3.5.2 Lack of access to adequate healthcare resulting in a reliance upon traditional remedies

Participants discussed how difficulties accessing healthcare led them to use traditional remedies:

“*We apply home remedies mostly because the hospital is not nearby.”* (FGD 2)

These access barriers were seen as promoting a reliance upon traditional remedies:

“*Medicines are not available when we get injured so we use home remedies. Home remedies can help.”* (FGD 4)

Conversely, some participants expressed their preference and confidence in traditional remedies which were rooted in superstitious and fatalistic beliefs. This was particularly seen in the use of ‘mantras' (prayers):

“*A mantra is done by the one who knows how to do it. Mantras can heal.”* (FGD 6)

Some participants expressed concerns about the risk of death if treated at a hospital. However, no discussion about how delayed treatment, due to longer distances could contribute to mortality:

“*Hospital treatment is of no use. People will die if we take them to hospital. Mantras are better than anything.”* (FGD 5)

However, treatment failure was also seen to occur when administered by a spiritual healer:

“*If a hand is broken we apply bamboo, egg, mash dal. We apply the paste to the hand along with a cloth and tie. When the bone is healed we also apply root of a type of lemon, turmeric and cow butter. We leave it like this for 1 week. If the hand is still not healed then try the same procedure again. If the Sipalu (healer) is good then the hand will go back to normal. But if it is not fixed properly by the one who is tying the hand, then the hand won't work properly and you cannot reach your head with your hand.”* (FGD 4)

## 4 Discussion

The fixed physical backdrop of the mountainous terrain in conjunction with gendered labor divisions, socio-economic pressures, and cultural beliefs force people into hazardous work with rudimentary tools, perpetuating the risk of certain injury. These factors do not operate in isolation, but intersect to create a layered vulnerability, particularly amongst female PCGs who bear the dual burden of domestic responsibilities and hazardous work. Despite recognizing the risks and being fearful when performing such tasks, PCGs prioritized household survival over personal safety. This illustrates the intersection of poverty, geography, gender, and limited agency to prevent certain injuries from occurring.

Participants contrasted Mugu to urban settings where they felt the risk of injury was lower. Available data (12) identifies that fewer injuries occur in urban areas compared to rural settings. However, participants did not consider that there will be different injury profiles associated with urban environments. This “unrealistic optimism about life events” due to optimism bias (31) where people might reject their present circumstances because they overestimate potential benefits elsewhere, arguably idealized urban locations as having limited injuries. This comparison between rural and urban areas has been documented in existing research (7, 20, 32).

Our findings identify that in addition to topography, infrastructure of the home itself compounds the risk of associated injury. The use of ladders and flat rooftops to beat hay represents a significant injury hazard, increasing the likelihood of falls (20). These identified architectural risks are reflective of findings in other districts in the Karnali region of Nepal (14, 51). Traditional cooking practices posed further risks, in particular using open fire cooking or rudimentary stoves on the floor located in the main living space (33). In addition to cooking, stoves and open fires are used to warm the home in colder months, and children and older adult people gather near the fire for warmth, increasing their risk of burns. These injury hazards mirror broader findings from rural and resource limited settings, where burns result from cooking practices which are shaped by environmental constraints and cultural traditions (20, 33–38, 52). Conversely, the home was also a place where participants felt they could exert a level of control over injuries, unlike outdoor environments. This dichotomy between home and external environments suggests a potential gap in injury prevention strategies, highlighting the need to consider both environmental and sociocultural factors when strategies are developed.

Participant discussion of their perceived agency to prevent injury was intimately connected to the gendered division of labor in Mugu. For instance, this division compels women to juggle hazardous agricultural work, home tasks, and childcare responsibilities. These responsibilities, compounded by malnutrition and physical weakness, arguably increased their vulnerability to associated injuries. Such insights help identify how the gender stratification of labor, when combined with rural isolation, poverty, and limited healthcare access, create multiple overlapping challenges that further heighten female PCG's exposure to certain injuries. In Mugu, about a quarter of its population is aged below 10 years (4), and PCGs reported a reliance upon older siblings to supervise younger children while they work. In addition, the mountainous topography and being sparsely populated means that children of all ages have to walk to school on their own or with older siblings. This results in inadequate supervision, leading to injuries to children, a finding consistent with existing literature (20, 33, 39, 40). PCGs recognize the injury risks associated with inadequate supervision, but view sibling supervision as the safest of limited options available.

A limited ability to exercise control over injury arguably fuels fatalistic beliefs of injuries as fated or a result of karma (20, 41). Attributing injuries to external forces which PCGs felt unable to influence, potentially functioned as a coping strategy, relieving their own accountability for the occurrence of injury to themselves or their children (20, 39, 42). However, this discourse which normalizes the inevitability of injury can be said to perpetuate the idea that few prevention measures can be taken, which may limit the development and implementation of policy or community-based interventions which aim to reduce injury risk.

Cultural beliefs in conjunction with economic constraints, geographical inaccessibility to formal healthcare, and lack of familiarity and mistrust of healthcare services, shape injury prevention and treatment behaviors (43, 44), as reflected in the use of traditional remedies. If wounds are slow to heal, these injuries are thought to be caused by spirits, which is why it can be argued that using traditional remedies which are grounded in superstitious beliefs offers a logical response. Conversely, the use of traditional remedies could be suggested to be a consequence of a lack of medical treatments and resources available in formal healthcare. In addition, whilst traditional remedies are accessible, they can make injuries worse, resulting in negative long term health outcomes (38, 45).

## 5 Strengths and limitations

This study was conducted in 2017 and focused on only one district in Nepal. While the data may seem dated, the injury risk in Mugu remains largely unchanged due to persistent environmental challenges linked to the fixed topography. As such, this study provides valuable baseline insights into PCGs perceptions of unintentional injuries that continue to hold relevance to inform current interventions in Mugu and neighboring mountainous districts.

To minimize the risk of cultural subtleties being misunderstood or overlooked, the first author collaborated closely with Nepali research assistants during data collection to ensure that the study was contextually grounded. Translation was used and therefore there is a possibility that some nuances in participant responses may have been lost or altered during this process. To mitigate this, a Nepali research assistant and co-author was present during the FGDs acted as transcriber and translator of the audio recordings.

Only 5% of FGD participants were male, which arguably means that men's perspectives on injury and injury prevention are missing. However, the selection criteria for participants was not based upon sex, but based upon being a primary PCG of a child or children aged 10 years or below. Therefore, the 5% inclusion of men in the study can be said to be reflective of the sex of PCGs in Mugu. However, to explore this further, future research could explore men's perspectives on injuries and injury prevention.

## 6 Conclusion

This study identifies a complex interplay of determinants contributing to reported high rates of unintentional injury in Mugu. Geographical factors including mountainous terrain and isolated location, coupled with inadequate infrastructure, contribute significantly to injury risk. However, as the topography of Mugu is fixed, environmental and structural factors are largely out of the control of PCGs. In response, socio-cultural and fatalistic beliefs have evolved, which regard injury as an inevitable and unavoidable part of daily life. Such beliefs appear prevalent in female PCGs, whose multiple roles in and outside the home expose themselves and the children in their care to daily risks of injury. This normalization of injury reinforces the perception of limited prevention options, compounded by inadequate access to quality healthcare, which were reported to have adverse economic and health impacts for the household.

At the time of conducting this study, Mugu experienced a significant international labor migration of young men seeking employment as migrant laborers abroad, a trend reflective of broader socio-economic shifts in Nepal. Whilst remittances from migrant workers may increase household income, and in some cases improve access to resources, this demographic shift also places considerable strain on those left behind, particularly women. Women in Mugu assume additional responsibilities, including managing agricultural work, household tasks, and looking after children. These expanded roles exacerbate PCG's physical and mental strain, increasing the risk of injury to themselves and the children in their care. However, despite these challenges, female PCGs are well-positioned to play a critical role in injury prevention interventions, provided these strategies acknowledge their existing workloads. Therefore, future interventions should address socio-cultural changes, such as international labor migration, and the impact men leaving to work abroad can have upon injury, childcare mechanisms to increase child supervision, increased risk assessment skills to reduce unnecessary risk taking, and engaging communities to prioritize local safety needs.

The launch of Nepal's constitution in 2015 marked a significant turning point, with the subsequent devolution of power to local governments creating opportunities to implement interventions tailored to local, municipality level needs. This political shift provides a promising opportunity to embed interventions within local systems. This ensures that they are responsive to the unique socio-cultural and geographical injury challenges of the community, and are appropriate, accessible and feasible within the constraints of PCG's current environment. Such initiatives may include the promotion of safer working practices, basic first aid skills, and active child supervision. This could empower PCGs to reduce injury risks by fostering a sense of agency and equipping them with practical prevention strategies.

There is a need for interventions in rural areas to focus on injury risks specific to the context, and tackle environmental, infrastructural, economic and cultural determinants of injury; including local perspectives of PCGs when developing interventions (46). Recognizing the normalization of injury as a central challenge, future research could integrate participatory approaches to develop and evaluate interventions which support a shift to injury prevention in settings like Mugu. Utilizing existing mechanisms such as that of Mother's Groups led by Female Community Health Volunteers (FCHV) could support with this shift (47, 48). The study reported here formed part of a wider research piece, which fed into the design and development of an mHealth intervention and face-to-face injury prevention training, that aimed to highlight the preventability of injuries to PCGs (Weatherburn, manuscript in preparation)^4^. By using participatory activities, such as transect walks, adapted risk matrices, and the creation of safety songs based on local “deuda's”, the community was able to reframe injury as something preventable rather than inevitable. These methods actively engaged participants in identifying injury risks and creating culturally and contextually appropriate solutions, enabling a shift to injury prevention which fostered a collective sense of responsibility and agency in injury prevention in Mugu.

At the policy level, structural improvements are essential, including the development of physical infrastructure such as building good quality roads, cementing foot trails, and installing lighting. By integrating structural reforms within PCG focused interventions, future initiatives have the potential to address both the immediate and long term challenges of unintentional injury in Mugu.

## Data Availability

The raw data supporting the conclusions of this article will be made available by the authors, without undue reservation.

## Footnotes

^1^Bikram Sambat (BS) is the official Nepali calendar, which is approximately 56.7 years ahead of the Gregorian calendar. Therefore 2079 BS corresponds to the year 2022/23.

^2^The Chulesi is a traditional knife of Mugu. The blade is used in the upright position with the person sitting down and with their legs either side of the blade.

^3^Due to the sub-alpine geographic location, limited grass is able to grow which means people have to depend on trees to collect leaves for cattle.

^4^Weatherburn K. *Unintentional injury prevention in rural Nepal*. London: King's College London. (unpublished).
